# Gender Differences in Psychological Outcomes Following Surf Therapy Sessions among U.S. Service Members

**DOI:** 10.3390/ijerph18094634

**Published:** 2021-04-27

**Authors:** Lisa H. Glassman, Nicholas P. Otis, Betty Michalewicz-Kragh, Kristen H. Walter

**Affiliations:** 1 Leidos, Naval Health Research Center, 140 Sylvester Road, San Diego, CA 92106-3521, USA; lisa.h.glassman.ctr@mail.mil (L.H.G.); nicholas.p.otis.ctr@mail.mil (N.P.O.); 2 Health and Behavioral Sciences Department, Naval Health Research Center, 140 Sylvester Road, San Diego, CA 92106-3521, USA; 3 Health and Wellness Department, Naval Medical Center San Diego, 34800 Bob Wilson Drive, San Diego, CA 92134, USA; betty.michalewiczkragh.civ@mail.mil

**Keywords:** physical activity, exercise, gender differences, sex differences, surf therapy, military, depression, anxiety, affect, pain

## Abstract

Surf therapy is increasingly being used as an intervention to address various health problems, including psychological symptoms. Although recent research supports the positive impact of surf therapy on psychological outcomes, it is unclear whether these outcomes differ between men and women. This study compared changes in depression/anxiety (Patient Health Questionnaire-4), positive affect (Positive and Negative Affect Schedule), and pain (Numerical Pain Rating Scale) between U.S. service men and women (*N* = 74) during six weekly surf therapy sessions. Overall, participants reported decreased depression/anxiety (*p* < 0.001) and increased positive affect (*p* < 0.001), but no change in pain rating following each session (*p* = 0.141). Significant gender differences were found in the magnitude of changes in depression/anxiety (*B* = −1.01, *p* = 0.008) and positive affect (*B* = 4.53, *p* < 0.001) during surf sessions, despite no differences in pre-session scores on either outcome. Women showed greater improvements in depression/anxiety and positive affect compared with men—an important finding, given that surfing and military environments are often socially dominated by men. Future research is needed to replicate these findings in other samples, extend this research to other underrepresented populations, and identify barriers and facilitators of the sustainable implementation of surf therapy across populations.

## 1. Introduction

The use of surfing as a therapeutic intervention to address psychological symptoms has increased significantly over the past several years [1,2]. Surf therapy programs operate around the world and are often offered as an adjunctive intervention to standard treatments to support psychological health and well-being. As the interest in surf therapy has increased, so too has research examining associated outcomes. Studies across several populations support the positive benefits of surf therapy on psychological functioning, psychosocial health, and physical strength [1].

U.S. active duty service members may derive particular benefit from surf therapy, given an emphasis on physical readiness and high rates of psychological symptoms in the military [3], as well as significant stigma against seeking traditional treatments in this population [4,5]. Initial research evaluating psychological outcomes among U.S. active duty service members following adjunctive surf therapy has shown positive benefits. In one study, six weekly surf therapy sessions resulted in significant improvements in posttraumatic stress disorder (PTSD; among individuals with probable PTSD), anxiety, depression, and positive and negative affect [6]. Research among veterans shows similar outcomes following surf therapy, including significant improvements in depression, PTSD, and self-efficacy [7,8,9]. In addition to examining program outcomes, Walter and colleagues (2019) examined psychological changes within individual surf therapy sessions; results demonstrated that positive affect significantly increased, and depression/anxiety decreased over the course of each surf therapy session. Service members also reported high levels of satisfaction with the surf therapy program [6]. Collectively, these findings provide initial support for the use of surf therapy as an adjunctive intervention resulting in psychological benefits and high satisfaction ratings among active duty service members and veterans [2,6]. However, surf therapy has yet to be directly compared to either traditional or alternative therapies; as such, more research is needed to establish the efficacy of surf therapy as well as the nuances of surf outcomes across diverse populations [2].

Although surf therapy research has demonstrated promising findings for the psychological health of service members and veterans, research has only begun to explore whether benefits are conditional on various factors. For example, recent research suggests that surf therapy outcomes may be moderated by psychological comorbidity status [10]. In particular, service members with comorbid (co-occurring) PTSD and major depressive disorder (MDD) demonstrated greater benefits following surf therapy sessions than those with either PTSD or MDD alone. Gender may be another important moderator of psychological outcomes following surf therapy. Although the Department of Defense (DoD), the Department of Veterans Affairs (VA), and service-related, non-profit organizations have implemented several surf therapy programs across the U.S., the sport of surfing remains socially dominated by men [11,12,13,14]. Despite an increasing female presence in the sport, women may feel marginalized in these gendered spaces, and their experience of surfing may be different from that of men [13,14,15,16]. These considerations may be particularly important both in therapeutic contexts, where it is critical to create a safe space where women can be vulnerable [17], and in exercise settings, where men and women may engage in, and respond to, exercise in different ways depending on self-efficacy, social support, body image, motivation, and subjective norms (such as gender typing) [18,19]. Although preliminary research suggests that surf therapy can offer a safe, non-judgmental environment for healing [20], more nuanced research is needed in this area.

Additional research in the military population would be particularly beneficial. Women operating within a male-dominated U.S. military culture and male-dominated surf environment may be particularly vulnerable to marginalization, which requires special effort and attention to mitigate [21,22,23]. Research on the unique experiences of female surfers and service members, especially as their numbers increase in both domains, is critical to ensure the sustainability of the sport in the future, as well as to ensure equal access to the benefits of surf therapy [24].

To our knowledge, there has only been one empirical examination of gender differences in psychological outcomes of surf therapy. The study found similar changes in “positive outlook” among male and female children and adolescents who participated in a 6-week surf therapy program in the United Kingdom [25]; however, these findings were based on non-validated instruments. In addition, in the study, girls were less likely than boys to complete the surf program, but no explanation for this difference was given. There have not been any empirical investigations into gender differences in surf therapy outcomes among adults, and, in fact, gender differences research across other sport therapeutics is similarly limited. Research in this area may be particularly important within the active duty population, given that military experiences and psychological outcomes resulting from service can differ by gender. For example, female service members are more likely than their male counterparts to experience military sexual trauma and early life stressors, as well as to be diagnosed with MDD [26,27,28,29].

Our study addressed this significant gap in the current surf therapy literature by examining gender differences in psychological outcomes among service members during the 6-week Naval Medical Center San Diego (NMCSD) Surf Therapy Program. The objective of this study was to examine gender differences in changes in depression/anxiety, positive affect, and pain rating over the course of individual surf sessions, as well as potential differences in the trajectory of these symptoms across the 6-week program. Although our analyses were exploratory in nature, we hope that our findings can be used to improve the sustainability of surf therapy and inform adaptations to surf therapy programs that will improve outcomes among underrepresented and understudied populations in general, and for women in particular. This study is a secondary data analysis of the main surf therapy program findings published elsewhere [6].

## 2. Materials and Methods

### 2.1. Participants

Study participants (*N* = 74; men, *n* = 41; women, *n* = 33) were U.S. active duty service members who were seeking treatment within the NMCSD Surf Therapy Program as part of their standard medical care. Participants are referred to the Surf Therapy Program with either a physical (e.g., neck or back pain) or psychological condition (e.g., depression, anxiety); among those with referral data available (*n* = 58), 79% of participants had a psychological condition as their primary referral diagnosis, while 21% had a physical condition. See Table 1 for additional demographic information. Those who had previously participated in the program or who had not received standard departmental medical clearance were ineligible for participation. (For additional information about study participants, the program, and procedures, please see Walter et al., 2019.)

### 2.2. Program

The Surf Therapy Program is offered as an optional component of standard medical care delivered to U.S. active duty service members at NMCSD. It is designed to rehabilitate those with physical or psychological conditions through the use of outdoor sports. The Surf Therapy Program operates in 6-week cohorts, with one 3–4 h session per week. Each cohort includes approximately 20 members. Each surf therapy session is a medical appointment. Each service member is paired with a volunteer surf instructor from the local community who works with the service member on a 1:1 basis. All volunteer instructors are vetted through the Armed Forces Young Men’s Christian Association. Volunteers receive training regarding patient safety, mental health (e.g., behaviors/symptoms associated with PTSD), and program policies and procedures by medical providers at NMCSD. All equipment is provided (e.g., surfboards, wet suits), and surf sessions take place on a public beach in Southern California. Consistent with standard operating procedures, prior to each session, service members have the option of attending an hour-long yoga class provided by the program.

### 2.3. Procedure

All study participants provided voluntary, written informed consent. Once enrolled, study participants completed brief self-report questionnaires before and after each surf session, in addition to a larger pre-program questionnaire containing demographic, service, and symptom information. Service members who did not wish to participate received surf therapy as usual and did not complete any study assessments. All study procedures were approved by the NMCSD Institutional Review Board.

### 2.4. Measures

**Pre–post-session assessments.** Before and after each surf therapy session, participants completed brief measures of depression/anxiety symptoms, positive affect, and pain rating. Data from these within-session assessments were used for the main study analyses and compared by gender.

***Depression/anxiety symptoms.*** Depression/anxiety was assessed before and after each surf therapy session using the 4-item Patient Health Questionnaire (PHQ-4) [30]. The PHQ-4 includes two depression items from the 8-item PHQ (PHQ-8) [31] and two anxiety items from the 7-item Generalized Anxiety Disorder (GAD-7) scale [32]. Each item is scored from 0 to 3; the scale’s summed response range is 0–12, with higher scores indicating greater self-reported depression/anxiety symptom severity. Internal consistency across session assessments was α = 0.834–0.935.

***Positive affect.*** Before and after each session, positive affect was assessed using the Positive Affect Schedule (PAS), a subscale of the Positive and Negative Affect Schedule (PANAS) [33]. This 10-item self-report subscale asks participants to rate their positive emotions over the last few hours on a scale from 1 to 5. Responses on the PAS are summed to create a total range of 10–50, with higher scores indicating greater positive affect. Internal consistency was excellent across all session assessments (α = 0.923–0.971).

***Pain rating.*** Pain rating was measured before and after each session using the Numerical Pain Rating Scale (NPRS) [34]. The NPRS is a single-item self-report measure with a scoring range of 0–10. Higher scores indicate higher perceived levels of physical pain.

**Pre-program questionnaire.** A self-report pre-program questionnaire was used to assess patient demographics, military characteristics, concurrent treatment, and baseline mental health symptom severity. In addition to characterizing the sample, select measures were used as between-subjects factors (e.g., gender) in analyses.

***Pre-program depression symptoms.*** Pre-program depression symptoms were measured using the PHQ-8. Each item is scored on a 0–3 rating scale, where the total scale summary range is 0–24 and higher scores indicate greater self-reported depression symptoms. Internal consistency was α = 0.874 at the pre-program assessment.

***Pre-program anxiety symptoms.*** Pre-program anxiety symptoms were measured using the GAD-7. Individual items are scored from 0 to 3, and severity scores are created by summing item responses (α = 0.909). The scale range is 0–21; higher scores indicate greater self-reported anxiety symptom severity.

***Pre-program positive and negative affect.*** Affect was measured prior to the start of the program using the PANAS. This 20-item scale can be divided into two 10-item subscales that assess positive (PAS) and negative affect (NAS). The PAS has been described above in its use as a session measure; its reliability at pre-program was α = 0.892. The NAS is similarly summed, with a scoring range of 10–50, where higher scores indicate greater self-reported negative affect (α = 0.897).

***Pre-program pain rating.*** Pain rating was measured prior to the start of the program using the NPRS, which has been described above for its use in the session assessments.

### 2.5. Data Analysis

Analyses compared men and women on within-session changes in depression/anxiety (PHQ-4), positive affect (PAS), and pain rating (NPRS) scores while accounting for potential confounding variables (i.e., concurrent treatment, yoga attendance, and week of session). Multilevel modeling (MLM) was used to best account for missing data and correlated error due to repeated measures both within participants and across time. Three separate models were used to examine changes in depression/anxiety, positive affect, and pain rating, respectively. Additionally, initial descriptive analyses compared relevant pre-program characteristics by gender, using chi-square and independent sample *t*-tests to test for differences. All analyses were completed in IBM SPSS Statistics Version 25 (IBM, Armonk, NY, USA).

Multilevel models used restricted maximum likelihood to account for missing data. Random effects were specified as follows: The intercept was random by subject; time (i.e., pre- to post-session) was crossed with weekly instances of session (week × time), with its slopes as random by subject; and the covariance matrix was set to unstructured. For repeated effects, time was crossed (week × time) and repeated by subject, and the covariance matrix was set to first-order autoregressive. These specifications allowed for appropriate clustering of data, as well as meaningful interpretation with consideration for the lowest Akaike information criterion (i.e., model fit). Specifically, the crossing of time within random effects (by subject) accounted for the random variation at each unique time point combination for an individual. The crossing of time within repeated effects captured how residuals were correlated both within session and across each weekly time point with respect to each individual.

In the first step, a base model was run as previously specified, with time (pre-session [0] to post-session [1]) as a fixed effect. In step two, level two independent variables were added as fixed effects: gender (0 = man, 1 = woman), concurrent treatment (0 = none, 1 = enrolled in concurrent treatment; e.g., psychiatry, psychotherapy), yoga attendance by session (0 = did not attend that day, 1 = did attend that day), and week of session (1–6). In the third and final step, interactions were added: time × gender, time × concurrent treatment, time × yoga, and time × week. This study was not adequately powered to test a three-way interaction of time × week × gender.

## 3. Results

Study sample (*N* = 74) characteristics, presented by gender, are shown in Table 1. Approximately 45% (*n* = 33) of the sample were women. The two gender groups differed in age (*M*_men_ = 30.2, *SD* = 8.4; *M*_women_ = 26.2, *SD* = 5.6; *p* = 0.023), but otherwise, no significant differences were found for any demographic or military characteristics, treatment utilization variables, or pre-program symptom scores. Means and standard deviations of all primary psychological variables are shown in Table 2.

### Pre- to Post-Session Analysis

Initial base models showed a significant change in outcome variables over time (*p*s < 0.001). Second step models (i.e., main effects only) revealed non-significant *p*-values for all independent variables except week and current treatment for the pain outcome. All independent variables were retained for final interaction models on the bases of theory, model fit, and implications of their interactions with time.

Final MLM results are presented in Table 3. The main effect of time (pre- to post-session) was significant for PHQ-4 (*B* = −2.40, *p* < 0.001) and PAS (*B* = 6.27, *p* < 0.001) but not NPRS (*p* = 0.141). That is, across all participants, on average, depression/anxiety decreased and positive affect increased, while pain remained unchanged over the course of a session. Despite starting with statistically similar scores at pre-session assessments (*p* = 0.804), women had greater decreases in depression/anxiety scores during sessions than men *(B* = −1.01, *p* = 0.008). Similarly, despite similar scores between genders at session onset (*p* = 0.316), women showed larger improvements in positive affect scores from pre- to post-session than men *(B* = 4.53, *p* < 0.001). The amount of change per session did not differ across sessions for depression/anxiety and positive affect, as indicated by non-significant time × week interactions for both outcomes (*p*s = 0.504–0.753). As noted previously, this study was not powered to test a three-way interaction to examine whether the amount of session change across weeks varied by gender. Neither concurrent treatment nor yoga attendance were predictive of changes in any outcomes examined, overall or differentially across sessions, suggesting that neither impacted depression/anxiety and positive affect over the course of a surf therapy session.

## 4. Discussion

Surf therapy is an emerging intervention, with growing evidence supporting its use for improving psychological symptoms. With increasing rates of psychological illness in the U.S. military [3], and significant military stigma against seeking standard psychological treatments [4,5], research examining the use of complementary intervention like surf therapy in the military population is critically important. Further, investigating how underserved or underrepresented populations respond to surf therapy may help to elucidate the nuanced impact of these interventions and how they can best be implemented to yield the greatest benefit across populations. Within the U.S. military, women remain a special population that must be appropriately considered.

Our findings are in line with prior research [6,7,8,9] and support the psychological benefits of surf therapy among individuals with psychological symptoms. Both men and women who participated in the study experienced significant increases in positive affect and decreases in symptoms of depression/anxiety over the course of individual surf therapy sessions, highlighting psychological benefits for both genders. When the effect of gender on outcomes was examined, we found that women experienced greater benefits than men following individual surf sessions. Women reported both greater decreases in depression/anxiety and greater improvements in positive affect. Interestingly, there were no effects of surf therapy on pain ratings for either gender; this may be because pain was rated on a single 0–10 scale, pre-session scores were generally low, or perhaps because changes in pain during surf sessions were variable across participants (e.g., pain may have improved for some [35] but not for others).

Study findings are important given the potential gender-related disparities in the sport of surfing and within the U.S. military as a whole. The observed gender differences in effects of surf therapy may be explained by several important factors. First, women may respond better than men because they experience less stigma around obtaining therapeutic interventions [36,37,38]. Women also have greater verbal affective fluency [39]; even in a physically demanding intervention such as surf therapy, there are numerous opportunities for conversation and self-expression with other participants and staff. Additionally, women in the U.S. military may find traditional care at DoD hospitals stigmatizing and uncomfortable; this has been reported among female veterans within the VA system [40,41]. As such, they may also be particularly responsive to supportive environments provided outside of military treatment facilities. Lastly, it is also possible that women and men might differentially experience feelings of self-efficacy, self-competence, or other psychosocial constructs in response to surfing, thus impacting psychological outcomes like affect or anxiety. Previous literature suggests that gender differences in these psychosocial constructs are associated with differences in engagement in, and response to, exercise [18,19], with self-efficacy acting as a stronger predictor among women than men. Considering that the NMCSD Surf Therapy Program contains elements that promote self-efficacy—connection, autonomy, skills-building, and positive group norms [42]—sessions may have been particularly impactful for women and further amplified in the context of surfing, where the modern culture of the sport in many countries (including the U.S.) is male-dominant [11,12,13,14,16]. Taken together, these factors regarding stigma, treatment setting, and psychosocial response may contribute to the greater demonstrated benefits of surf therapy for women in our study.

This study’s findings are encouraging, but more research is needed on surf therapy among female service members to replicate the benefits of the intervention because our analyses were exploratory. If women truly do respond better than men, research should also examine what men may need in order to enhance their response to surf therapy as well. Overall, the examination of gender differences in response to complementary therapeutic environments is a critically understudied area of research but is necessary to ensure the effectiveness and sustainability of the intervention.

This study has several notable limitations. The study demographic questionnaire only assessed binary self-reported gender (i.e., man, woman). Our findings may have been more nuanced if we had examined sex assigned at birth in addition to an expanded list of current gender identity. Future research should also examine the role of intersectionality on surf therapy outcomes because individuals can strongly associate with more than one identity (e.g., gender, sexual orientation, race/ethnicity, physical ability) that may influence their experiences in, and therapeutic response to, surfing. Further, all psychological domains assessed in this study relied on self-report questionnaires, which are subject to inherent limitations. Additionally, data collected for the original study did not include surf instructor characteristics (e.g., gender), prior participant surf experience (although participants could not have previously participated in the Surf Therapy Program), or environmental variables (e.g., wave height, weather) that may have differentially influenced an individual’s response to surf therapy. Future studies should consider collecting these data and examining the influence on outcomes. However, random variation due to many of these factors was statistically addressed using random effects for slope by participant (e.g., may capture surfing experience), as well as random effects for day (represented by week of session; may capture variance in surfing conditions). The original study also did not collect follow-up data from participants after participation in the Surf Therapy Program, which would inform whether the gender differences in surf therapy outcomes were sustained over time. Lastly, most participants received other therapeutic interventions in addition to surf therapy. As such, it is difficult to differentiate the unique impact of surf therapy versus a combination of surf therapy and other psychological interventions. To address this, we statistically controlled for variance in our outcomes due to these confounding factors (including the optional yoga session offered prior to each surf therapy session). Further, study assessments were conducted immediately before and after surf therapy sessions, increasing confidence that the changes observed are predominately due to changes experienced during surf therapy sessions rather than other factors.

There are also several unique and important strengths to this study. First, we examined outcomes of a complementary therapeutic intervention, surf therapy, for which both popularity and empirical support are increasing. We also examined outcomes in the unique population of U.S. service members, who have not been adequately studied in the current body of research. Second, examining gender differences in surf therapy allowed for exploration of outcomes among women, who remain an underrepresented and understudied population within the U.S. military and with respect to sport-related therapies. The examination of gender differences across sport therapeutics requires additional research attention to ensure that women (and other genders) receive comparable benefits to men, especially within environments that are traditionally dominated by men or are gender-typed as masculine. Third, the sample was diverse regarding female representation within the U.S. military. Specifically, the sample consisted of approximately 43% women, whereas women comprise approximately 17% of the U.S. military [43]. Lastly, study procedures used in the original study allowed for the assessment of the immediate benefits of surf therapy by including assessments before and after each session, and generalizability is high due to the naturalistic methodology (Walter et al., 2019).

### Future Directions

Based on these promising findings, research must turn to the sustainability of surf therapy across different populations. It is necessary to determine how to increase the accessibility of a therapeutic intervention that requires a specific environment (e.g., ocean, river, large lake, wave pool) and considerable resources (i.e., purchasing surfboards and wet suits) [44]. Non-profit organizations, along with the U.S. DoD and VA healthcare systems, have increased access to surf therapy; however, this remains an effort that primarily focuses on those who live near the ocean or who are members of specific populations (e.g., service members, veterans). More resources are needed to support the uptake of surf therapy among historically underserved populations who may significantly benefit from physically, environmentally, and socially oriented therapeutics but may experience barriers to participation, such as cost, stigma, treatment unavailability, and lack of inclusive social or cultural environments. Similarly, future research could focus on testing and deploying novel technologies (e.g., wave generators in pools) that can improve access to surf therapy across groups that otherwise would not have geographical access to this intervention. Continued research on the effectiveness of surf therapy among varying populations (e.g., women, people with physical disabilities, members of different racial/ethnic groups)—with a focus on how to improve outcomes in these populations, if necessary—is also critically important to ensuring that this therapy is delivered in an equitable environment and modified as necessary for specific groups. Lastly, examining potential barriers to engaging in surf therapy, such as stigma, structural barriers, or perceived inability, would also be beneficial to ensuring sustainability. Although preliminary research suggests that outdoor therapeutic programs may reduce stigma about mental health and mental health injuries [45], additional research focusing on psychological barriers to surf therapy can guide implementation efforts in the future.

## 5. Conclusions

Our results suggest that both male and female U.S. service members experience psychological benefits following individual surf therapy sessions. However, female service members, compared with their male counterparts, experienced greater improvements in symptoms of depression/anxiety, as well as positive affect, over the course of surf therapy sessions. These findings are aligned with other research indicating that women experience better outcomes following mental health treatment compared with men; this study extends these findings to sports-oriented therapy. This is particularly important in surf therapy, considering that the sport of surfing is traditionally a male-dominated environment that can marginalize individuals with other gender identities. Future research should focus on replicating these findings in larger samples and extending this research to other underrepresented populations. Further, research encouraging the sustainability of surf therapy, including examining access and barriers to care, is necessary and will be critical in enhancing the broader implementation of this intervention.

## Figures and Tables

**Table 1:** Pre-program sample characteristics.

Characteristic	Total Sample (*N* = 74)	Men (*n* = 41)	Women (*n* = 33)
	*n* (%)	*n* (%)	*n* (%)
Service branch ^a^			
Navy	55 (76.4)	–	–
Marine Corps and Coast Guard	17 (23.6)	–	–
Rank ^a^			
E1–E4	32 (45.1)	–	–
E5–E9	33 (46.4)	–	–
Officer	6 (8.4)	–	–
Concurrent treatment			
Any treatment	56 (76.7)	29 (72.5)	27 (81.8)
Psychiatry	18 (24.7)	9 (22.5)	9 (27.3)
Psychotherapy	33 (45.2)	15 (37.5)	18 (54.5)
Recreational therapy ^b^	29 (39.7)	13 (32.5)	16 (48.5)
Primary referral diagnosis ^a^			
Depression	13 (22.4)	–	–
PTSD	13 (22.4)	–	–
Anxiety	6 (10.3)	–	–
Adjustment disorder	5 (8.6)	–	–
Other psychological condition	9 (15.5)	–	–
Neck or back pain	6 (10.3)	–	–
Other physical condition	6 (10.3)	–	–
	*M* (*SD*)	*M* (*SD*)	*M* (*SD*)
Age, years	28.4 (7.5)	30.2 (8.4) *	26.2 (5.6) *
Sessions attended			
Surf therapy	4.4 (1.7)	4.6 (1.6)	4.1 (1.8)
Yoga	1.6 (1.9)	1.8 (2.0)	1.5 (1.7)
Pre-program measures			
PHQ-8	12.4 (5.9)	12.4 (6.7)	12.4 (5.0)
GAD-7	12.7 (5.7)	12.5 (5.8)	12.9 (5.6)
PAS	24.0 (8.7)	23.8 (8.3)	24.2 (9.2)
NAS	24.2 (9.6)	23.5 (9.1)	25.0 (10.2)
NPRS	2.7 (2.3)	3.1 (2.6)	2.4 (1.9)

Note. E = enlisted rank; GAD-7 = 7-item Generalized Anxiety Disorder scale; NAS = Negative Affect Schedule; NPRS = Numerical Pain Rating Scale; PAS = Positive Affect Schedule; PHQ-8 = 8-item Patient Health Questionnaire; PTSD = posttraumatic stress disorder. Because concurrent treatment categories are not mutually exclusive, the sum across treatment categories is greater than 100%. For other columns, totals may not sum to sample numbers or percentages due to missing data. Asterisks indicate significant difference between gender groups. ^a^ Breakdowns by gender for these variables cannot be provided due to small cell sizes that might be identifying. ^b^ Recreational therapy consists of other programs within the Naval Medical Center San Diego Health and Wellness Department, such as other sports, music, art, and animal-assisted therapy. * *p* < 0.05.

**Table 2:** Means and standard deviations of psychological outcomes at session assessment time points.

Time Point		PHQ-4			PAS			NPRS		
		*n*	*M*	*SD*	*n*	*M*	*SD*	*n*	*M*	*SD*
Session 1	Pre-session 1	72	6.19	3.27	72	24.44	9.46	71	2.76	2.28
	Post-session 1	71	2.37	2.31	71	32.73	9.92	70	2.55	2.18
Session 2	Pre-session 2	67	5.78	3.22	67	24.42	10.21	64	3.27	2.48
	Post-session 2	65	2.98	2.87	65	32.55	11.31	63	3.17	2.65
Session 3	Pre-session 3	59	5.73	3.10	59	25.19	10.13	58	3.10	2.61
	Post-session 3	58	2.52	2.74	58	35.93	9.73	57	2.53	2.34
Session 4	Pre-session 4	53	5.89	3.21	53	25.34	10.21	53	3.19	2.35
	Post-session 4	52	2.56	2.29	52	35.17	9.58	51	2.79	2.31
Session 5	Pre-session 5	46	5.35	3.45	46	26.57	11.47	45	3.04	2.40
	Post-session 5	46	2.24	2.81	46	34.54	11.62	46	2.60	2.39
Session 6	Pre-session 6	24	6.29	3.36	24	26.38	11.48	24	3.04	2.54
	Post-session 6	24	2.00	2.34	24	36.75	10.18	24	2.96	2.40

Note. NPRS = Numerical Pain Rating Scale; PAS = Positive Affect Schedule; PHQ-4 = 4-item Patient Health Questionnaire.

**Table 3:** Final multi-level analyses examining pre–post-session scores for depression/anxiety, positive affect, and pain rating.

	PHQ-4		PAS		NPRS	
Variable	Estimate	95% CI	Estimate	95% CI	Estimate	95% CI
Intercept	6.21 ***	[4.85, 7.56]	25.92 ***	[20.95, 30.89]	1.64 **	[0.50, 2.77]
Time	−2.40 ***	[−3.39, −1.41]	6.27 ***	[3.40, 9.14]	0.35	[−0.12, 0.81]
Week	−0.06	[−0.23, 0.11]	0.23	[−0.34, 0.80]	0.16 *	[0.04, 0.28]
Concurrent treatment	−0.13	[−1.52, 1.25]	−1.61	[−6.76, 3.55]	1.51 *	[0.32, 2.69]
Yoga	−0.21	[−0.90, 0.48]	0.78	[−1.43, 2.99]	0.42 *	[0.03, 0.81]
Gender	1.44	[−1.00, 1.29]	−2.18	[−6.48, 2.12]	−0.67	[−1.66, 0.32]
Time × week	−0.04	[−0.27, 0.20]	0.25	[−0.49, 0.99]	−0.09	[−0.25, 0.07]
Time × concurrent treatment	−0.90	[−1.83, 0.03]	1.90	[−0.83, 4.62]	−0.21	[−0.67, 0.25]
Time × yoga	0.38	[−0.35, 1.11]	−1.39	[−3.55, 0.77]	−0.50 **	[−0.84, −0.16]
Time × gender	−1.01 **	[−1.75, −0.27]	4.53 ***	[2.37, 6.70]	−0.30	[−0.66, 0.06]

Note. CI, confidence interval; NPRS = Numerical Pain Rating Scale; PAS = Positive Affect Schedule; PHQ-4 = 4-item Patient Health Questionnaire. * *p* < 0.05. ** *p* < 0.01. *** *p* < 0.001.

## Data Availability

Neither the data nor the materials have been made available on a permanent third-party archive; requests for the data or materials can be sent via email to the lead author at lisa.h.glassman.ctr@mail.mil.

## Disclaimer

I am a military service member or employee of the U.S. Government. This work was prepared as part of my official duties. Title 17, U.S.C. §105 provides that copyright protection under this title is not available for any work of the U.S. Government. Title 17, U.S.C. §101 defines a U.S. Government work as work prepared by a military service member or employee of the U.S. Government as part of that person’s official duties. Report No. 21-18 was supported by the U.S. Navy Bureau of Medicine and Surgery under work unit no. N1600. The views expressed in this article are those of the authors and do not necessarily reflect the official policy or position of the Department of the Navy, Department of Defense, nor the U.S. Government. The study protocol was approved by the Naval Medical Center San Diego Institutional Review Board in compliance with all applicable Federal regulations governing the protection of human subjects. Research data were derived from an approved Naval Medical Center San Diego Institutional Review Board protocol, number NMCSD.2016.0032.
